# Supplementary figures and images for: Integration of cooperative and opposing molecular programs drives learning-associated behavioral plasticity
Source: PLoS Genet. 2023 Mar 27;19(3):e1010650. doi: 10.1371/journal.pgen.1010650 (PMC10079226; doi:10.1371/journal.pgen.1010650)

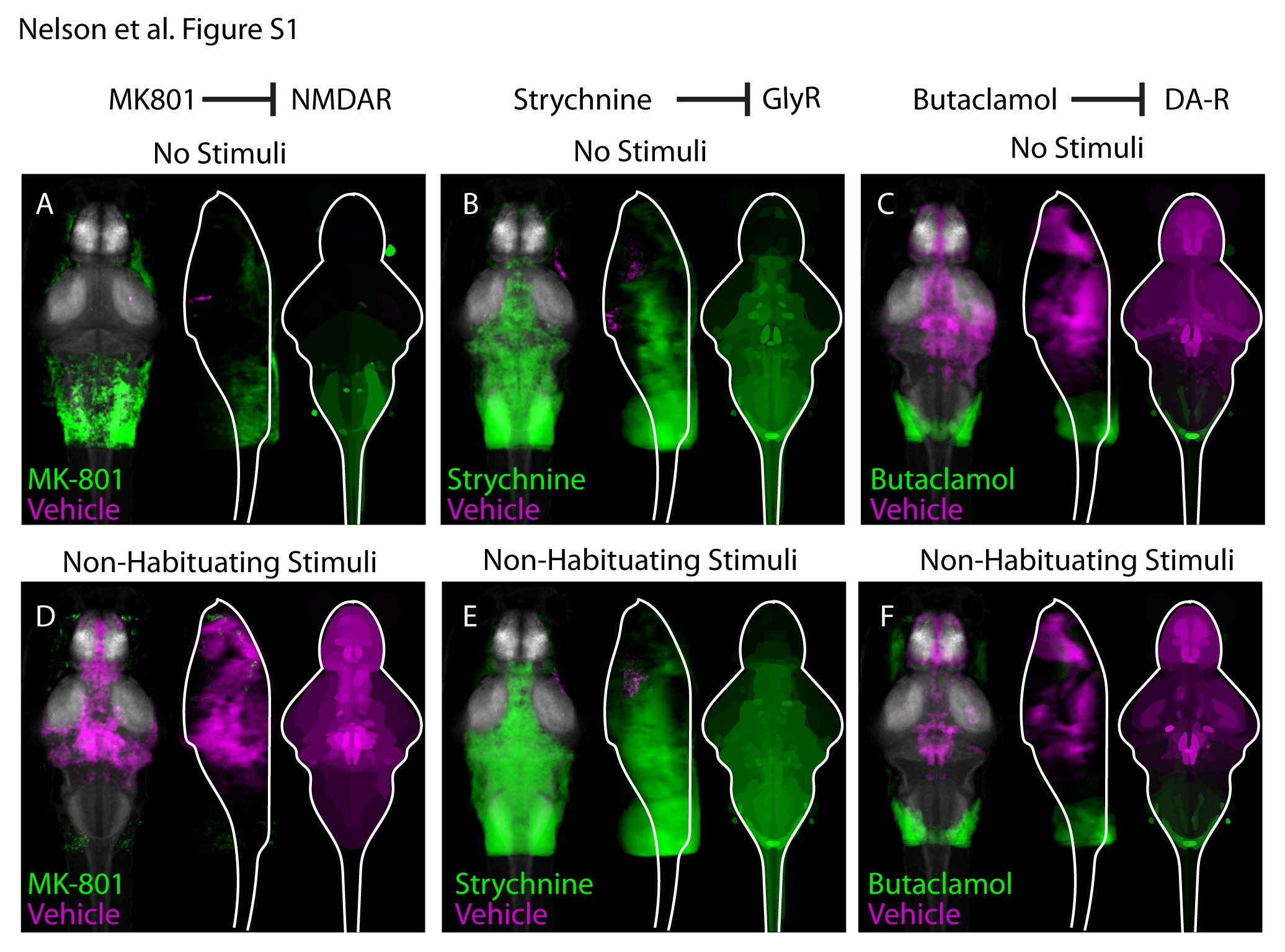

Supplement: S1 Fig — (A-C) Regions upregulated by the specified drug treatment under “No Stimulus” conditions are indicated in green; regions downregulated are indicated in magenta. (D-F) Regions upregulated by the specified drug treatment under “Non-Habituating Stimuli” conditions are indicated in green; regions downregulated are indicated in magenta. In all images, the left panel is a summed z-projection of the whole-brain activity changes. The middle panel is a summed x-projection of the whole brain activity changes. The right panel is a z-projection of the analyzed MAP-map. Molecular targets of pharmacological agents are indicated with diagrams above each column. Note that the patterns of neuronal activity induced by a given pharmacological agent are relatively consistent across stimulation condition (i.e. “no stimuli”, vs. “non-habituation stimuli”, vs “habituating stimuli” in Fig 1K–1M). Moreover, although all pharmacological agents reduce habituation learning, patterns of neuronal activity are highly dissimilar between individual pharmacological treatments. (TIF) [file pgen.1010650.s001.tif]

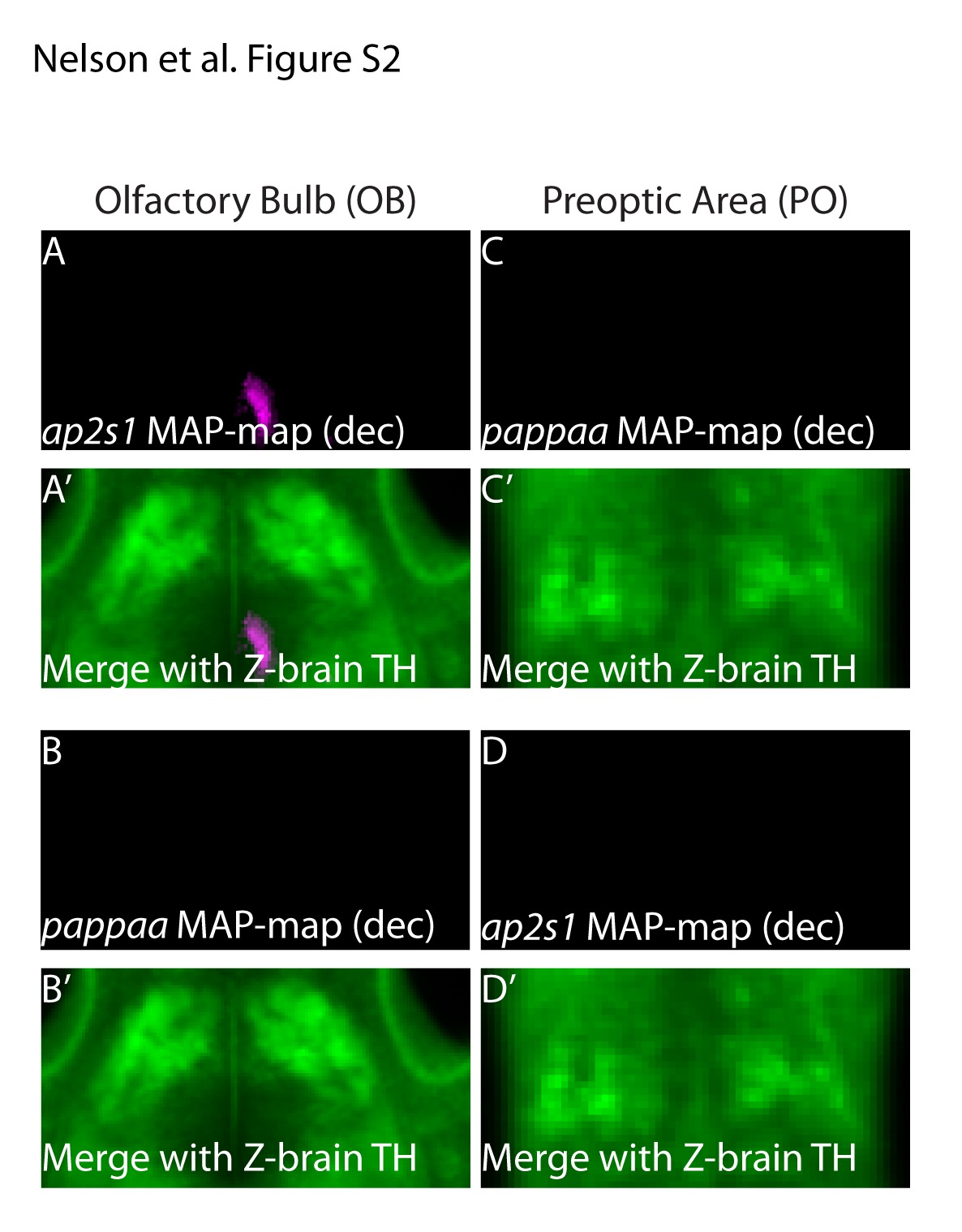

Supplement: S2 Fig — (A-D’) Regions with reduced activity in mutants relative to siblings are indicated in magenta. These signals are merged with the Z-brain-registered Th stain in green showing olfactory bulb dopaminergic neurons in figures A-B’ and preoptic dopaminergic neurons C-D’. Fig 7 shows increased activity in olfactory bulb dopaminergic neurons in ap2s1 and pappaa mutants and preoptic dopaminergic neurons in pappaa mutants. Here we show that these same regions are largely devoid of pixels showing reduced activity in mutants relative to siblings according to our MAP-maps. No areas of reduced activity are identified, except medially, outside the Th-stained area of the olfactory bulb in A-A’. These data are consistent with ap2s1 and pappaa upregulating rather than downregulating dopamine neuron activity within these brain areas. (TIF) [file pgen.1010650.s002.tif]

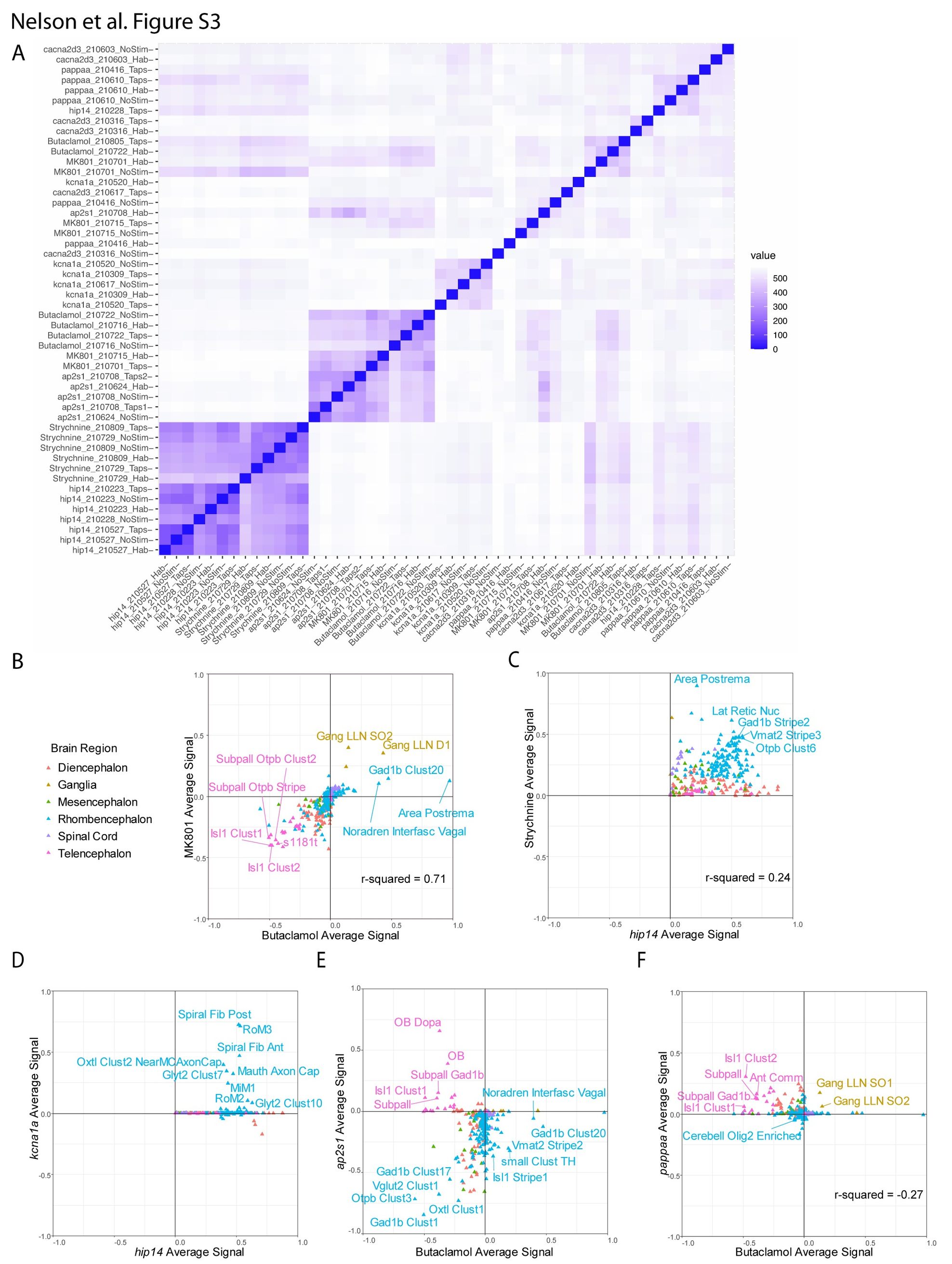

Supplement: S3 Fig — (A) Heat map indicating replicability across stimulus conditions for each mutant and drug condition. Column and row labels indicate genotype or drug treatment and stimulus condition (i.e. No Stimuli = NoStim, Non-Habituating Stimuli = Taps, Hab = Habituating Stimuli). Note an intermingled cluster containing Butaclamol and MK-801. (B-F) Plots of pairwise comparisons between drugs and or genotypes. Color legend in (B) applies to all. R-square values are indicated when p<0.05. (B) Plot indicating positive correlation between MK-801 and Butaclamol signal changes. (C) Plot indicating a weak positive correlation between hip14 and Strychnine signal changes. (D) Plot showing correlated changes in the rhombencephalon between hip14 and kcna1a. (E) Plot showing 3 populations in Butaclamol vs. ap2s1 changes. Largely telencephalic regions, upregulated in ap2s1 mutants and downregulated by Butaclamol; largely rhombencephalic regions, upregulated by Butaclamol and downregulated in ap2s1 mutants; and a large number of regions downregulated by both manipulations. (F) Plot showing signal changes in pappaa as compared to Butaclamol. Multiple telencephalic as well as diencephalic regions to a lesser degree, are anti-correlated (up-regulated in pappaa mutants but downregulated in Butaclamol). Brain region abbreviations in (B): s1181t = Telencephalon—S1181t Cluster, Gang LLN SO2 = Ganglia—Lateral Line Neuromast SO2, Gang LLN D1 = Ganglia—Lateral Line Neuromast D1, Noradren Interfasc Vagal = Rhombencephalon—Noradrendergic neurons of the Interfascicular and Vagal areas. Brain region abbreviations in (C): Lat Retic Nuc = Rhombencephalon—Lateral Reticular Nucleus. Brain region abbreviations in (D): Spiral Fib Post and Ant = Rhombencephalon—Spiral Fiber Neuron Posterior and Anterior clusters, Mauth Axon Cap = Rhombencephalon—Mauthner Cell Axon Cap. Brain region abbreviations in (E): DO = Telencephalon—Olfactory bulb dopaminergic neuron areas, OB = Telencephalon—Olfactory Bulb, Subpall Gad1b = [file pgen.1010650.s003.tif]

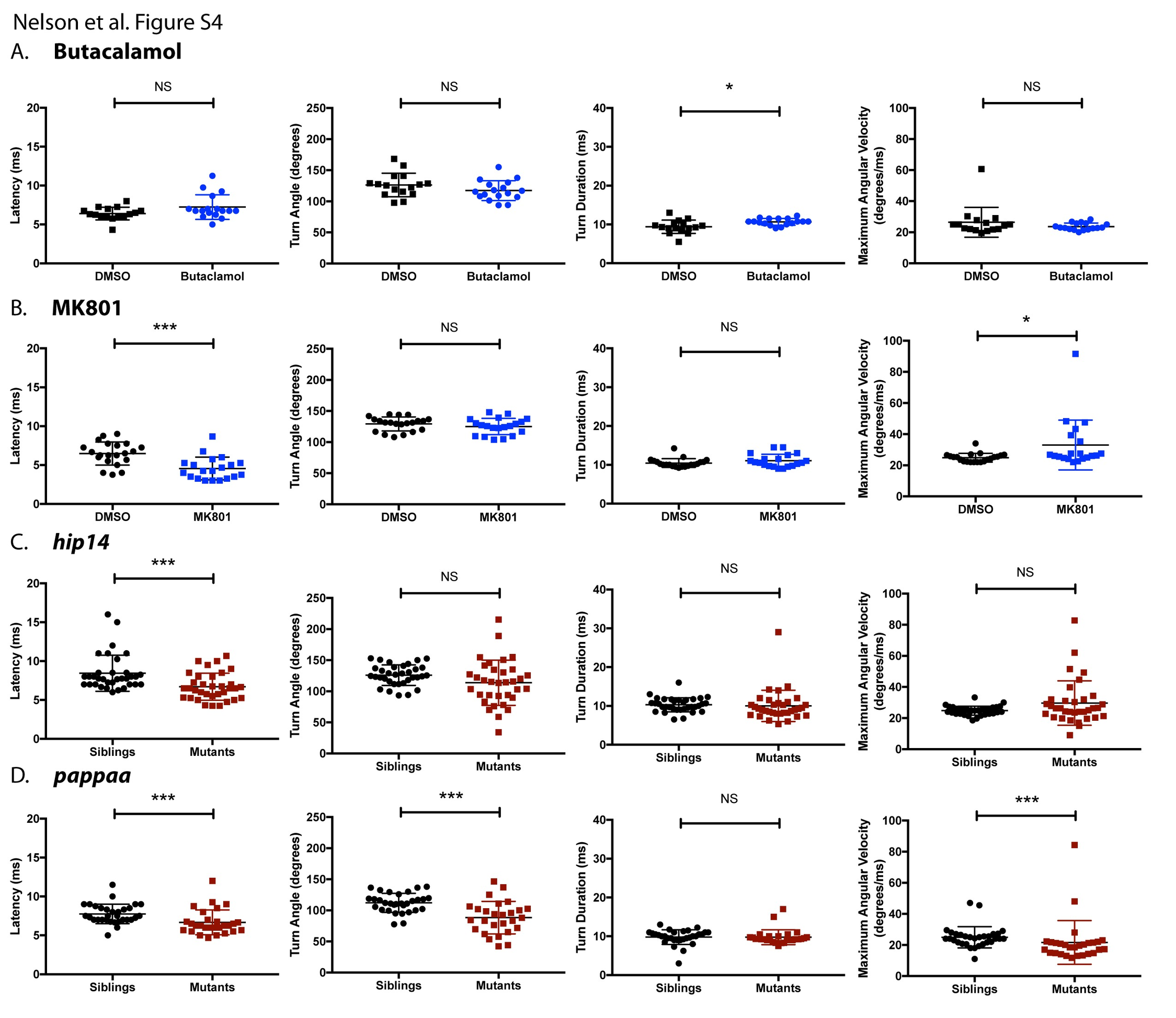

Supplement: S4 Fig — Kinematic parameters for MK801, Butaclamol, hip14, and pappaa are reported (for ap2s1, cacna2d2, and kcna1a see references 33,29,32 respectively. Bonferroni-adjusted p-values for unpaired t-tests are reported for normally distributed data, and Bonferroni-adjusted p-values for Mann-Whitney tests are reported for data that are not normally distributed. NS = Not significant. (A) Butaclamol significantly increases turn duration during acoustic startle performance n = 17 Butaclamol-treated, n = 16 DMSO-treated, Bonferroni-adjusted p-value for Mann-Whitney test p = 0.044. (B) MK801 significantly reduces turn latency and significantly increases maximum angular velocity during acoustic startle performance. Latency: n = 20 MK801-treated, n = 21 DMSO-treated, Bonferroni-adjusted p-value for Mann-Whitney test p = 0.0004. Maximum angular velocity: n = 20 MK801-treated, n = 21 DMSO-treated, Bonferroni-adjusted p-value for Mann-Whitney test p = 0.0468. (C) Mutations in hip14 significantly reduce turn latency during acoustic startle performance. n = 34 hip14 mutants, n = 34 hip14 siblings, Bonferroni-adjusted p-value for Mann-Whitney test p = 0.0008. (D) Mutations in pappaa significantly reduce turn latency, turn angle, and maximum angular velocity during acoustic startle performance. Latency: n = 28 pappaa mutants, n = 31 pappaa siblings, Bonferroni-adjusted p-value for Mann-Whitney test p = 0.0008. Turn Angle and maximum angular velocity: n = 28 pappaa mutants, n = 31 pappaa siblings, unpaired t-test p<0.0001. (TIF) [file pgen.1010650.s004.tif]
